# 2756. Activity of Aztreonam-avibactam and Ceftazidime-avibactam against United States Isolates Producing β-lactamases (2020–2021)

**DOI:** 10.1093/ofid/ofad500.2367

**Published:** 2023-11-27

**Authors:** Mariana Castanheira, Valerie Kantro, Timothy Doyle, Rodrigo E Mendes, Helio S Sader

**Affiliations:** JMI Laboratories, North Liberty, Iowa; JMI Laboratories, North Liberty, Iowa; JMI Laboratories, North Liberty, Iowa; JMI Laboratories, North Liberty, Iowa; JMI Laboratories, North Liberty, Iowa

## Abstract

**Background:**

β-lactamase (BL) –producing isolates are widespread and threaten the use of β-lactams. We evaluated the activity of ceftazidime-avibactam (CAZ-AVI) and aztreonam-avibactam (ATM-AVI) and comparators against common BLs detected in US hospitals.

**Figure:**
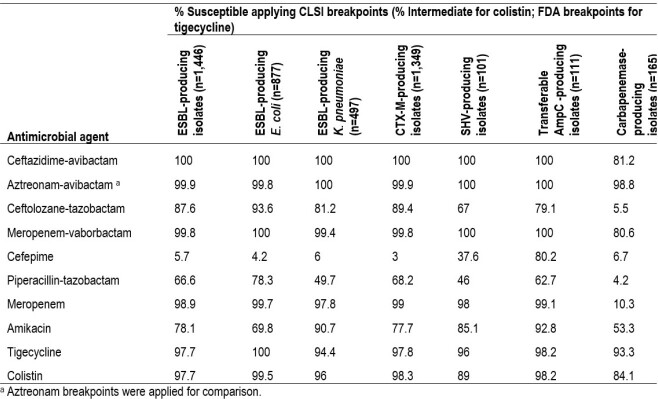

**Methods:**

A total of 21,853 Enterobacterales (ENT) isolates collected during 2020–2021 were susceptibility (S) tested by reference broth microdilution methods. Isolates submitted to whole genome sequencing were: (1) *Escherichia coli* (EC; n=1,013) and *Klebsiella pneumoniae* (KPN; n=621) displaying MIC values ≥ 2 mg/L for at least 2 of the following: ceftazidime, ceftriaxone, aztreonam, or cefepime; (2) *Enterobacter cloacae* (n=452) and *Citrobacter* spp. (n=169) displaying MIC values ≥ 16 mg/L for ceftazidime and/or ≥ 2 mg/L for cefepime; and (3) ENT (n=240) displaying elevated carbapenem (meropenem and/or imipenem) MIC results at > 1 mg/L.

**Results:**

CAZ-AVI inhibited all ESBL-producers (n=1,446; without carbapenemases) including EC, KPN, and isolates producing CTX-M enzymes (Table). Meropenem-vaborbactam (MEV) inhibited 99.8–100% and ceftolozane-tazobactam (CT) inhibited 67–93.6% of these isolates. ATM-AVI inhibited > 99.4% (using an aztreonam alone breakpoint) of the isolates regardless of the ESBL type or organism. Meropenem S rates against ESBLs ranged from 97.8 to 99.7%. Among other classes, amikacin and tigecycline were the most active agents, inhibiting 78.1% and 97.7% of the ESBL-producing isolates. A total of 97.7% of the isolates had intermediate colistin MIC values. All isolates carrying transferrable AmpC genes were S to CAZ-AVI, ATM-AVI, and MEV and 99.1% were susceptible to meropenem, but only 79.1% were S to CT. These included 82 CMY-producers. Among carbapenemase producers (n=165), CAZ-AVI, ATM-AVI, and MEV susceptibility rates were 81.2%, 98.8% and 80.6%. The only comparator displaying activity against these isolates was tigecycline (93.3% susceptible).

**Conclusion:**

Avibactam combinations were active against common BL-producing isolates from US hospitals, including carbapenemase-producing isolates for which therapeutic options are limited. ATM-AVI was the most active agent against carbapenemase-producers when applying the aztreonam breakpoints for comparison.

**Disclosures:**

**Mariana Castanheira, PhD**, AbbVie: Grant/Research Support|Basilea: Grant/Research Support|bioMerieux: Grant/Research Support|Cipla: Grant/Research Support|CorMedix: Grant/Research Support|Entasis: Grant/Research Support|Melinta: Grant/Research Support|Paratek: Grant/Research Support|Pfizer: Grant/Research Support|Shionogi: Grant/Research Support **Valerie Kantro, BA**, AbbVie: Grant/Research Support|Pfizer: Grant/Research Support|Shionogi: Grant/Research Support **Timothy Doyle, MS**, AbbVie: Grant/Research Support **Rodrigo E. Mendes, PhD**, AbbVie: Grant/Research Support|Basilea: Grant/Research Support|Cipla: Grant/Research Support|Entasis: Grant/Research Support|GSK: Grant/Research Support|Paratek: Grant/Research Support|Pfizer: Grant/Research Support|Shionogi: Grant/Research Support **Helio S. Sader, MD, PhD, FIDSA**, AbbVie: Grant/Research Support|Basilea: Grant/Research Support|Cipla: Grant/Research Support|Paratek: Grant/Research Support|Pfizer: Grant/Research Support|Shionogi: Grant/Research Support

